# Checkpoints in Adenoviral Production: Cross-Contamination and E1A

**DOI:** 10.1371/journal.pone.0023160

**Published:** 2011-08-03

**Authors:** Dagmar J. Haeussler, Alicia M. Evangelista, Joseph R. Burgoyne, Richard A. Cohen, Markus M. Bachschmid, David R. Pimental

**Affiliations:** 1 Vascular Biology Section, Boston University Medical Center, Boston, Massachusetts, United States of America; 2 Myocardial Biology Unit, Boston University Medical Center, Boston, Massachusetts, United States of America; University of South Florida College of Medicine, United States of America

## Abstract

Adenoviruses are widely used for overexpressing proteins in primary mammalian cells. Incorporation of the early viral gene, E1A, or viral cross-contamination can occur during amplification, and identification of these products is crucial as the transcription of unwanted genetic material can impact cell function and compromise data interpretation. Here we report methods for evaluation of contaminating adenovirus and E1 viral DNA.

## Introduction

The generation and use of adenoviruses (Ad) in physiological research has become a routine procedure due to their ability to increase protein expression in primary mammalian cells that cannot be efficiently transfected with plasmid DNA. Creating and amplifying an Ad is a time and labor intensive process; therefore it is necessary to determine as early as possible that the gene of interest (GOI) is free of mutations and is not contaminated with another coding sequence. This is particularly pertinent when creating more than one Ad at a time using site directed mutagenesis (i.e. wild type and mutant), as it is not possible to verify the authenticity of the final adenovirally expressed protein using standard immunoblot techniques. Although it is common practice to screen the Ad cosmid DNA for errors, in our experience the stage at which Ad are most susceptible to unwanted contamination, and therefore the most important to screen, is during the initial formation of a viral plaque. During this period of time a single contaminating viral particle, i.e. from aerosolization of another Ad used in the same sterile hood, or an Ad that is amplified in the same incubator as the single clones, is enough to cause contamination, which may replace expression of the intended GOI with that of the contaminating virus. We therefore propose routine evaluation of the initial viral plaques should be done using a simple PCR-based method and suggest routine decontamination of incubators used to amplify Ad.

**Figure 1:**
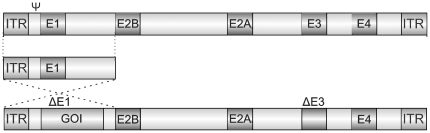
Homologous recombination of HEK293 Ad5 genomic DNA with the Ad construct. HEK293 cells contain bases 1–4345 of Human Ad serotype 5 genome, including the packaging signal (ψ) and E1 region. Overlapping Ad5 sequences in 2^nd^ generation Ad constructs can undergo single or double homologous recombination to yield replication competent Ad.

Refined vector systems, such as AdEasy, make Ad production a relatively straight forward procedure. This system is based on the human Ad serotype 5 genome and belongs to the so called 2^nd^ generation Ad constructs that omit the early viral genes, E1 and E3, but maintain the viral packaging signal and inverted terminal repeat (ITR) required for viral transcription. The benefits of using 2^nd^ generation Ad are that they can accommodate a GOI up to 7.5 kb. Using a bacterial plasmid containing left and right arm flanking homology domains, the GOI is inserted into the site of the disrupted E1A by homologous recombination. E1A is a viral transcription factor required for induction of early genes necessary for viral replication, including E1B and E2 [1], [2], progression of the cell cycle, and inhibition of apoptosis [2]. E3, though not technically required for viral replication, plays an important role in suppression of host immunity [3], [4]. In the absence of E1A and E3, the recombinant Ad can infect but not replicate in most cells. Since 2^nd^ generation Ad lack E1 and E3, rendering them non-replicative, they must be amplified in packaging cell lines that express E1 gene products. In HEK293 cells, which are transformed with bases 1–4355 (containing full-length E1) of sheared human Ad5 genome, the Ad with GOI insert can be packaged and amplified via E1A and E3 supplied *in trans*. However, because many vector systems maintain significant portions of the human Ad5 genome, single or double homologous recombination events between the transformed HEK293 cells and the viral cosmid can occur, resulting in the production of replication competent Ad contamination (Figure 1). Though rare in early passages of adenovirus, the probability of replication competent Ad contamination increases with large-scale amplification [5], a phenomenon that has led to the decline of HEK293 cells in adenoviral production for clinical usage but has not translated to the basic science laboratory. Even optimized protocols fail to warn about the possibility of recombination with the early viral genes and how to monitor the authenticity of Ad during the amplification process via a simple PCR method.

## Materials and Methods

Ad constructs were prepared and cesium chloride purification was performed according to the previously reported methods [6], [7].

### Isolation of Ad DNA and PCR for E1A

DNA was isolated from cesium chloride-purified Ad using proteinase K extraction with the RedExtract-N-Amp Tissue PCR kit (Sigma). Isolated Ad DNA was tested for the presence of E1A contamination using the following primer sets as reported by Lavine et al. and Kuwano et al. [8], [9]:

E1A nested outer primers: 5′-CTGCCACGGAGGTGTTATTACC-3′ and 5′-CTCAGGTTCAGACACAGGACCT-3′

E1A nested inner primers: 5′-GAACCACCTACCCTTCACGAACTG-3′ and 5′-GTGGCAGGTAAGATCGATCACCTC-3′.

E1A primer set: 5′-TATGCCAAACCTTGTACCGGAGGT-3′ and 5′-CCGGGGTGCTCCACATAATCT-3′

Nested PCR reactions were performed sequentially. All reactions were performed using REDExtract-N-Amp PCR Reaction Mix containing JumpStart Taq polymerase (Sigma Aldrich) using an Endurance TC-512 Thermocycler (Techne Inc.). Primers were added at a final concentration of 5 µM to 1× PCR Reaction Mix containing 4 µl DNA for a final volume of 20 µl. Nested PCR was performed with initial denaturation at 94 degrees for 3 mins followed by 30 cycles with denaturation for 1 min at 94 degrees, annealing for 45 secs at 63 degrees and extension for 1 min at 72 degrees. Final extension was held for 10 mins at 72 degrees. Confirmation of PCR products was performed using the E1A primer set and was performed with initial denaturation at 94 degrees for 3 mins followed by 30 cycles with denaturation for 1 min at 94 degrees, annealing for 30 secs at 60 degrees and extension for 45 secs at 72 degrees. PCR products were analyzed on a 1% agarose gel containing 200 ng/ml ethidium bromide (Bio-Rad) in 1× TAE buffer. Bands were visualized using the Bio-Rad Gel-Doc system.

### Isolation of Ad DNA and PCR for GOI

DNA was isolated from early amplifications by taking a small portion of the cell suspension before freeze/thawing, obtaining a cell pellet by centrifugation (5 min, 1000 rcf) and performing proteinase K extraction with the RedExtract-N-Amp Tissue PCR kit (Sigma).

Primer I (within affinity tag): 5′-CAAATGGGTCGGGATCTGTA-3′

Primer I (within pshuttle-CMV): 5′- ACCGTCAGATCCGCTAGAGA- 3′

Primer II (downstream GOI): 5′-GATCCGGTGGATCGGATAAA-3′

Primer III (Sequencing Primer): 5′-GATCCAGAACCATTTTGTG-3′

Primers were added at a final concentration of 0.5 µM to 1× PCR Reaction Mix containing 3–7 µl DNA (depending on viral titer). PCR reaction was performed with an initial denaturation at 94 degrees for 3 mins and annealing at 60 degrees for 3 min, followed by 30 cycles with denaturation for 1 min at 94 degrees, annealing for 30 secs at 60 degrees and extension for 30 secs at 72 degrees. Final extension was held for 10 mins at 72 degrees. 5 µl of PCR product was analyzed for size as described above. The remaining PCR reaction was processed with a PCR cleanup kit (Qiagen) and submitted for sequencing using the Tufts sequencing core facility.

## Results and Discussion

**Figure 2:**
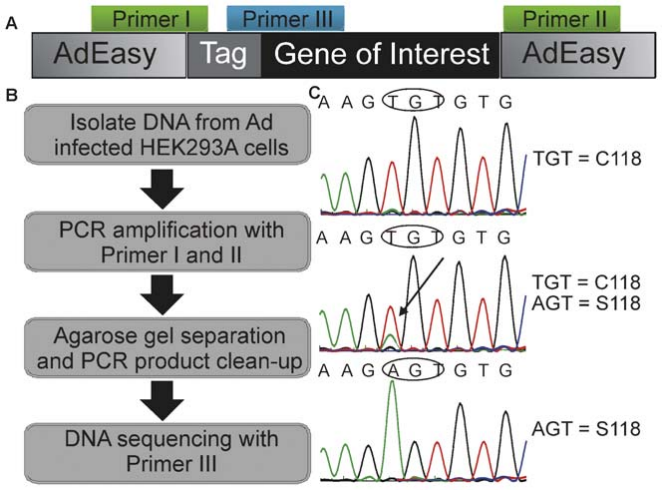
Contamination of Ad constructs during replication. (A) Primer locations for PCR of Ad and subsequent sequencing. Primer I and primer III ensure PCR of viral DNA by overlapping either with an affinity tag or part of the construct sequence. (B) Procedure used for sequencing Ad. (C) Sequencing results from early stage Ad amplification demonstrating contamination of the GOI.

To specifically amplify only GOI and not the HEK cell endogenous gene, we designed a set of PCR primers (Figure 2A), where Primer I binds either at the junction between the upstream vector and GOI sequence or, if available, within the affinity tag sequence of the GOI. Primer II binds at the sequence downstream of GOI and ensures amplification of only the Ad sequence. The PCR products were checked for specific amplification and size via agarose gel separation, and DNA sequencing services were used to determine the authenticity of the PCR product (Figure 2B). Depending on the size of GOI, one or more sequencing primers (Primer III) should be designed to cover the entire sequence. When using multiple primers, contiguous primers should be designed to generate overlapping sequences. By using this method we identified a contaminant during an attempt to create an Ad. In this particular case we found 3 different populations of Ad from the initial viral plaque. In Figure 2C, the chromatograms show 1) the desired mutation C118, 2) a mixed virus population of C118 contaminated with S118, and 3) a completely contaminated clone with S118. With this PCR-based method it is not only possible to detect cross-contamination but also accidental mutation that can happen while the virus is amplified from a single copy to up to 10^12^ viral particles per ml. This screening technique ensures that only viral clones with the correct DNA sequence are propagated and provides assurance that results obtained with Ad can be accurately interpreted.

**Figure 3:**
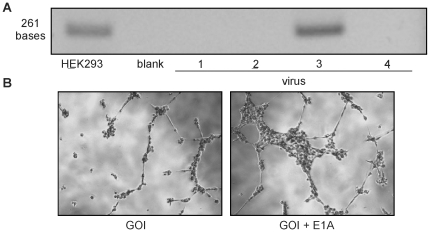
Detection and effects of E1A in HAEC. (A) DNA was extracted from cesium chloride purified Ad or HEK293 cells and PCR was performed with primers for E1A. Ad 3 shows E1A contamination. (B) VEGF-induced tube formation in HAEC infected with either Ad containing the GOI or Ad coding for the GOI and the contaminant E1A.

Following amplification in HEK293 cells, high titer virus can be purified using the cesium chloride density centrifugation method, which yields high purity viral stock. We propose routine screening of this stock for the presence of contaminating replication competent Ad. Traditional sequencing protocols for the viral genome rely on reverse transcription of mRNA produced in Ad transfected cells; however, using proteinase K extraction, we purified viral DNA and directly tested for E1A using PCR. We tested four viruses amplified in HEK293 cells and found E1A contamination present in one Ad stock (Figure 3A). Using the contaminated Ad and an Ad containing the same GOI but no E1A contamination, we infected human aortic endothelial cells (HAEC) and measured cellular migration using a tube formation assay in Matrigel-coated plates. Ad contaminated with E1A showed a marked increase in tube formation over the same virus without E1A (Figure 3B) in response to VEGF, demonstrating the possible confounding impact of E1A contamination in cell culture studies. Testing for E1A contamination after purification of Ad should be standard practice in order to ensure the integrity of results in Ad-transduced cells.

## References

[1] Nevins JR (1986). Control of cellular and viral transcription during adenovirus infection.. CRC Crit Rev Biochem.

[2] Wu L, Rosser DS, Schmidt MC, Berk A (1987). A TATA box implicated in E1A transcriptional activation of a simple adenovirus 2 promoter.. Nature.

[3] Schaack J, Bennett ML, Colbert JD, Torres AV, Clayton GH (2004). E1A and E1B proteins inhibit inflammation induced by adenovirus.. Proc Natl Acad Sci U S A.

[4] Ginsberg HS, Lundholm-Beauchamp U, Horswood RL, Pernis B, Wold WS (1989). Role of early region 3 (E3) in pathogenesis of adenovirus disease.. Proc Natl Acad Sci U S A.

[5] Lochmuller H, Jani A, Huard J, Prescott S, Simoneau M (1994). Emergence of early region 1-containing replication-competent adenovirus in stocks of replication-defective adenovirus recombinants (delta E1 + delta E3) during multiple passages in 293 cells.. Hum Gene Ther.

[6] Luo J, Deng ZL, Luo X, Tang N, Song WX (2007). A protocol for rapid generation of recombinant adenoviruses using the AdEasy system.. Nat Protoc.

[7] Adachi T, Pimentel DR, Heibeck T, Hou X, Lee YJ (2004). S-glutathiolation of Ras mediates redox-sensitive signaling by angiotensin II in vascular smooth muscle cells.. J Biol Chem.

[8] Lavine JA, Raess PW, Davis DB, Rabaglia ME, Presley BK (2010). Contamination with E1A-positive wild-type adenovirus accounts for species-specific stimulation of islet cell proliferation by CCK: a cautionary note.. Mol Endocrinol.

[9] Kuwano K, Nomoto Y, Kunitake R, Hagimoto N, Matsuba T (1997). Detection of adenovirus E1A DNA in pulmonary fibrosis using nested polymerase chain reaction.. Eur Respir J.

